# Modern Aspects of Phototherapy for Atopic Dermatitis

**DOI:** 10.1155/2012/121797

**Published:** 2011-12-15

**Authors:** Sonja Alexandra Grundmann, Stefan Beissert

**Affiliations:** Department of Dermatology, University of Münster, 48149 Münster, Germany

## Abstract

Phototherapy has still great importance in the treatment of atopic dermatitis, though costs, compliance, and long-term risks narrow its relevance. In spite of its long history, up to now, the therapeutic regimes are mostly empirical. Narrowband UVB und UVA1 are the most frequently applied regimens in atopic dermatitis with proven efficacy. However, even for these modalities randomized prospective and controlled studies are still pending. Advances in photoimmunology and molecular biology had demonstrated that phototherapy targets inflammatory cells, alters cytokine production, and has a significant antimicrobial effect within atopic skin. This paper summarizes the current literature on the different regimes of phototherapy and also discusses therapeutic modalities like photochemotherapy and extracorporeal photopheresis. These more complex regimes should be restricted to severe cases of atopic dermatitis, which are refractory to topical treatment.

## 1. Introduction

Atopic dermatitis is a common chronic relapsing inflammatory skin disease, which affects patients of all life decades. Therapy of atopic dermatitis is mainly based on topical therapies like moisturizers, corticosteroids, or calcineurin inhibitors and avoidance of trigger factors. Systemic anti-inflammatory treatment remains an option in severe cases. Though a beneficial effect of solar exposure has been appreciated for decades, phototherapy of atopic dermatitis has been largely empirical. Since the early twenties it was known that sea climate can improve atopic dermatitis and soon it was additionally recognized that atopic dermatitis improved during the summer season. In 1948, the helpful effects of UV radiation were studied for the first time by exposing patients to radiation emitted from carbon arc lamps [1]. From the 1970s on, fluorescent lamps were applied and in spite of more or less precisely defined emission spectra, some of these types of lamps are still in use. In the last years therapies like UVA1 and narrowband UVB (311 nm) gained importance not at least as a result of wide experience in other inflammatory dermatoses like psoriasis. In general, phototherapy is indicated in chronic stages of atopic dermatitis, except UVA1 which is also effective in acute flares [2]. However, phototherapy has to be a part of a comprehensive treatment plan, which has to consider some limitations. UV therapy requires special technical equipment and trained staff. Furthermore, patients have to be compliant to follow a therapy plan for 3–5 times a week up to 6–12 weeks. Some areas like hairy skin and skin folds are difficult to treat, which limits the efficiency. The advances in photoimmunology and molecular biology give explanation to the mode of action of different phototherapeutic regimes. Phototherapy targets inflammatory cells, alters cytokine productions and has a significant antibacterial effect. Nevertheless, the majority of concepts in phototherapy of atopic dermatitis are still empirical today. The lack of randomized controlled trials, which compare the different phototherapeutic regimens, still limits recommendations for the most appropriate phototherapeutic regimen. This paper deals with the main present modalities of UV therapy. Searches on phototherapy and atopic dermatitis were performed using Pubmed and cross-references of these publications.

## 2. UVB Phototherapy

### 2.1. Broadband UVB

UVB phototherapy (290–320 nm), as the “oldest” phototherapeutic regimen, has a long tradition in treating atopic dermatitis and started with the exposure of patients to carbon arc lamps. UVB fluorescent and mercury arc lamps made UVB regimens the therapy of choice for quite a long time and its efficacy was documented in several studies [3–5]. In one of the first studies, atopic patients (*n* = 17) were irradiated with broadband UVB (0.5–1.0 minimal erythema dose, MED) compared with visible light (each regimen applied to one-half of the body over 8 weeks). This led in the majority of patients to a complete healing of the lesions compared to visible-light-exposed areas. The same group examined also the therapeutic dose response to UVB (0.8 MED versus 0.4 MED applied to one-half of the body) for eight weeks (*n* = 24). However, although both UVB doses were found to be effective, no significant differences were found between the doses applied. This supports the fact that lower doses are equally effective compared with near erythemogenic doses [3]. In another study in 107 atopic patients, UVB was applied once daily for 4–19 days. A beneficial effect was observed in 93% of the cases as well as a significant corticosteroid-sparing effect [6]. However, the used irradiation device (Psorilux 9050) emitted also to a certain extend in the UVA range when looking at the reported emission spectra. In psoriasis, which is still the most frequent indication for UVB therapy, the comparison of the therapeutic spectra showed the highest efficacy in the range of around 313 nm [7, 8]. Subsequently, a narrowband (311–313 nm) UVB fluorescent lamp (Philips TL01) was introduced [9].

### 2.2. Narrowband UVB

Narrowband UVB is much less erythemogenic due to the exclusion of short wave lengthUVB irradiation. Data comparing the carcinogenic risks of narrowband UVB and broadband UVB are limited in humans [10]. In 2004, the skin cancer risk in 195 psoriasis patients treated with broadband or narrowband UVB phototherapy was surveyed retrospectively and did not provide evidence for a significant increased skin cancer risk during an observation period of ten years [11]. However, prospective longitudinal studies with prolonged follow-up periods are required. In a large cohort (*n* = 1908) a median number of 23 treatments did not result in an increased incidence of squamous cell carcinoma or melanoma (median followup 4 (0.04–13) years), though the risk of basal cell carcinoma was increased twofold. However, to determine the definite risk, definitely longer followup is essential as well [12]. Based on the human carcinogenesis action spectrum, the carcinogenic risk of narrowband UVB lamps is estimated 50% higher for equal erythemal doses than selected broadband lamps. An epidemiologic evidence is still pending [13], while in mice, narrowband UVB irradiation produced more malignant skin tumors [14]. Nevertheless, narrowband UVB is more effective in treating psoriasis and fewer treatments are needed to achieve remission, which might overall weigh up the higher carcinogenic risk by less exposure. In psoriasis, narrowband UVB is superior and has almost entirely replaced broadband UVB, which led to other indications including atopic dermatitis [15–20]. Several studies clearly demonstrate the effectiveness of narrowband UVB for treatment of atopic dermatitis and furthermore show that long-term benefit can be achieved by phototherapy. Due to worsening of itch and sweating especially during UVA/UVA-1 therapy, patients have been treated with air-conditioned narrowband UVB phototherapy three times weekly for 12 weeks (*n* = 21), which resulted in a 68% reduction in atopic dermatitis severity scores and a concomitant 88% reduction in topical corticosteoird use [17]. Even after six months 15 patients in this open study had long-term benefit. The activity of atopic dermatitis was markedly reduced after three weeks of irradiation with a cumulative dose of 9 J/cm^2^ narrowband UVB [18]. The effect of narrowband UVB has been also evaluated in children. The response was good to excellent in 80% (*n* = 40, mean age 11 years) whereas the most frequent adverse effects were erythema and xerosis [15, 20]. Jury et al. reported that 68% of children achieved minimal residual diseases after treatment (*n* = 25). However, it was not commented on the eczema severity, length of remission, or whether topical treatment was continued during treatment with narrowband UVB [21]. A six-year retrospective study (1999–2005) of narrowband UVB phototherapy for children with atopic dermatitis (*n* = 50) achieved in 40% of these patients severe eczema clearance or minimal residual activity; median length of remission was three months [22]. These patients were permitted to use topical steroids, so it is difficult to assess the effect of narrowband UVB alone. Recent studies confirmed narrowband UVB to be an effective and well-tolerated treatment modality in children [23, 24]. In conclusion, UVB 311 nm can be successfully used for the phototherapy for children with atopic dermatitis. Nevertheless, based on the potential long-term adverse events it should not be regarded as first-line treatment [25]. Since different forms of phototherapy including 8-methoxypsoralen bath-PUVA [psoralen plus UVA (PUVA)] are empirically efficient in atopic patients, it is important to compare them systematically. Narrowband UVB was compared to PUVA (each applied on the half of the body) in patients with severe atopic dermatitis (*n* = 12, three times weekly for six weeks). After cessation of phototherapy, a decrease in the mean baseline SCORAD score by 64% (PUVA) and 66% (narrowband UVB) was observed [17]. Hence, both regimens appear to be equally effective in atopic dermatitis. No acute severe adverse effects were reported. In a more recent randomized controlled trial narrowband UVB (*n* = 26), broadband UVA (*n* = 24), and visible light (*n* = 23) phototherapy has been applied twice weekly for 12 weeks. Narrowband UVB was demonstrated to be very effective in moderate-to-severe adult atopic dermatitis and remission lasted three months. Only moderate effects were noted for broadband UVA phototherapy [19]. However, this study did not demonstrate a significant corticosteroid-sparing effect by either irradiation regimens as has been reported before [17]. A therapy of oral short-term cyclosporin A for 4 weeks, followed by a washout phase of 4–6 weeks and consecutive narrowband UVB phototherapy (3 times/week, up to 2 months) has been reported to be effective in the treatment of severe atopic dermatitis [26]. However, long-term effects of this protocol has to be viewed very critically especially regarding its carcinogenic potential. A small trial by Legat et al. compared narrowband UVB to medium-dose UVA1 via half-side comparison in nine patients with chronic atopic dermatitis and confirmed the effect of narrowband UVB. A 40% reduction of clinical severity score (Costa score) under treatment with narrowband UVB and a better reduction of pruritus was documented while treatment with UVA1 did not achieve any statistically significant disease reduction [27]. However, more recent trials demonstrated narrowband UVB and medium-dose UVA1 to be equally effective in the treatment of moderate-to-severe atopic dermatitis [28, 29].

### 2.3. UVA/B Phototherapy

Combination phototherapy of UVA and UVB irradiations has quite a long history in atopic eczema [4, 30, 31]. It can be applied by using special tubes whose emission spectrum includes both ranges (e.g., Metec Helarium) or by combining UVA and UVB tubes simultaneously or in a subsequent manner. UVA/B therapy was preferentially used since UVA/B could be demonstrated to be superior to conventional broadband UVB in atopic dermatitis. Altogether in 48% of patients a complete remission was achieved with UVA/B phototherapy (*n* = 23) compared to only 27% in patients with UVB (*n* = 33) with an average of 5 irradiations per week over 4 weeks [32]. These results were confirmed by a later report. In 30 patients, a combination of UVA and UVB on one side of the body and UVB on the other was applied 3 times/week for a total of eight weeks. In this clinical evaluation, UVA/B treatment was reported to be superior for all scores including the pruritus score [4]. However, regarding the extent of the disease, no statistically significant differences were notable. A disadvantage of the combined irradiation is that it is impossible to dose UVA and UVB separately. In devices which are equipped with UVA and UVB tubes, both spectra can be dosed individually. UVA/B therapy was popular, because of its superiority to broadband UVB. Since the introduction of narrowband UVB and UVA1, UVAB therapy has lost some of its importance but is still in use. In children, a combination of UVB and UVA for atopic dermatitis resulted in 68.3% of the patients in a >70% reduction of the SCORAD index [31]. In adult patients with atopic dermatitis, an ongoing topical therapy with corticosteroids resulted in significant clinical improvement. Topical corticosteroids reduced the total UVB dose required and the duration of treatment without any overall difference on remission or side effects compared with UVA/UVB monotherapy [33].

### 2.4. UVA(-1) Phototherapy

In contrast to UVA/B, pure UVA therapy plays a rather minor therapeutic role. Conventional UVA fluorescent tubes have only a limited output and thus require relatively long exposure times to achieve biologically effective doses. UVA-1 devices, which cover the long wave length range from 340 to 400 nm, emit rather high doses in a reasonable amount of time [34–39]. The rationale for developing UVA-1 lamps was the assumption to reduce the adverse effects by omitting the UVA-2 part (320–340 nm), which is closer to the UVB range. Initially, these high power UVA-1 lamps were used to perform photoprovocations, consecutively, these lamps were also used for therapeutic purposes, because high doses of UVA-1 could be applied without inducing, sunburn reaction. It has been reported to be beneficial for patients with acute and recalcitrant atopic eczema [36, 37, 40, 41]. UVA-1 penetrates deeper into the skin than UVB and UVA-2, thus, higher doses can reach the dermis, and the superficial blood vessel plexus and cause biological effects [42, 43]. In the first pilot study of UVA-1 irradiation in atopic dermatitis, a single dose of 130 J/cm^2^ was given for 15 consecutive days [36]. This treatment regimen was compared to UVA/B irradiation (starting doses 30 mJ/cm^2^ UVB and 7 J/cm^2^ UVA, resp.). UVA-1 was significantly more effective compared to UVA/B therapy, as well in clinical scores and in the downregulation of eosinophilic cationic protein levels [44]. It was quite surprising and unexpected that all patients responded to UVA-1 therapy after only six exposures [37]. Several years later, a multicenter follow-up study with more patients revealed that UVA-1 high-dose therapy was superior in comparison with topical corticosteroids and UVA/B therapy [37]. However, the initially reported quick response of the previous study could not be reproduced [35]. UVA-1 high dose therapy is a valuable therapeutic option especially in acute severe atopic dermatitis but is by far not the standard treatment as it was promoted after the initial pilot study. Unfortunately, high power UVA-1 devices develop heat when applying these high doses and many atopic patients do not tolerate this. This led to the development of UVA-1 lamps, which filtered the infrared part, so-called “UVA-1 cold light”. The efficacy of this regimen was shown in a variety of studies. A medium-dose UVA-1 cold light (50 J/cm^2^/day for 15 days) induced a significant reduction of the SCORAD score and cytokine receptor levels in atopic eczema [39]. The clinical effect was still present after a one-month follow-up. However, after a three-month follow up a recurrence of symptoms could be noted [34]. These regimens gave rise to quite controversial opinions about extremely high doses, as medium-dose UVA-1 regimen was shown to be also of therapeutic benefit [34, 39]. As a consequence, a comparison of high-dose versus medium-dose UVA-1 irradiation was initiated. In a half-side comparison by Tzaneva et al. high-dose UVA-1 irradiation (130 J/cm^2^/day for 15 days) led to a 35%, medium-dose UVA-1 (65 J/cm^2^/day for 15 days) to a 28% decrease in the SCORAD score [38]. In another study, patients were randomized to receive either low-dose (20 J/cm^2^), medium-dose (65 J/cm^2^), or high-dose (130 J/cm^2^) UVA-1 [35]. It was found that the medium-dose and high-dose treatment regimens were superior as compared to the low-dose UVA-1 treated group of patients. However, there were no significant differences between the high-dose and the medium-dose groups and the tolerability was higher in the medium-dose group, which supports the concept that medium-dose UVA-1 is comparatively as effective as exposure to high-doses of UVA-1 for the treatment of patients with severe generalized atopic dermatitis. A medium dose of UVA-1 cold light (45 J/cm² five times weekly for 4 weeks) showed prolonged therapeutically effects concerning disease activity and quality of life [45]. However, until now, no state-of-the-art regimen could be designed relating to the optimal dose and the duration and frequency of the treatment. As long as controlled studies with large numbers of patients are lacking, preference should be given to medium-dose regimes. Partial body UVA-1 phototherapy is an option for localized and defined areas, which is proven to be successful in the treatment of dyshidrotic eczema of the palms and soles. In atopic dermatitis, medium-dose UVA-1 phototherapy induced in 10 out of 12 patients healing of the lesions (15 irradiation cycles) and no relapse occurred up to 3 months [46]. Therefore, local UVA-1 treatment appears to be an alternative option for the treatment of chronic dermatitis, however, comparative studies against other phototherapies including photochemotherapy are rarely reported [47].

One of the major mechanisms of UVA-1 is T lymphocyte apoptosis, as demonstrated by in vitro studies. CD4+ T cells are found in atopic lesions and these T cells undergo apoptosis upon UVA-1 exposure as demonstrated in situ [43]. The appearance of apoptotic T cells was followed by their depletion from cutaneous lesions, a reduction in the in situ expression of IFN-gamma by T cells and clearing of the atopic inflammation. Furthermore, UVA-1 is also able to modulate the balance between the antiapoptotic proteins as potent regulators of T-cell apoptosis [48–51]. Taken together, UVA-1 irradiation acts symptomatically by elimination of the inflammatory lymphocytic infiltrate.

## 3. Photochemotherapy

Photochemotherapy terms the combination of psoralens with UVA (PUVA) [52]. Psoralens (8-methoxypsoralen, 8-MOP) can be applied orally (systemic PUVA) or topically (bath- or cream-PUVA). Systemic PUVA has proven to be effective in severe atopic eczema, but relatively large numbers of exposures had to be given [53–59]. In several patients who did not continue topical application of corticosteroids, a rebound phenomenon occurred after termination of PUVA. PUVA has been used in dermatology for over 30 years and long-term safety data are available mostly for psoriatic patients. In psoriasis, long-term systemic PUVA is without doubt associated with the increased risk to develop skin malignancies. After 25 years, more than 50% of the patients with more than 400 treatments developed at least one squamous cell carcinoma. Almost one-third of the patients exposed to more than 200 treatments developed at least one basal cell carcinoma [60–62] and it is also known that the risk of malignant melanoma particularly in long-term therapy is higher as well (>250 treatments) [63–65]. In atopic dermatitis, long-term safety data have not been investigated so far. However, the potential risk must be weighed against the potency of other therapies. In spite of long-term follow-ups, which are essential in these patients, the performance of long-term PUVA for atopic patients is strongly discouraged. Systemic PUVA can occasionally cause side effects like nausea and vomiting, although this problem is much less common with 5-methoxypsoralen, which is no longer available. It is also less phototoxic, thus theoretically, 5-methoxypsoralen should be favored. Unfortunately, it is not approved in most countries and has been taken off the market recently. Other disadvantages of oral psoralens are the relatively long photosensitivity (elimination not before 8 hours after ingestion) and the necessity of eye protection. These problems can be avoided, when applying psoralens topically [55, 66]. For bath-PUVA, patients are bathing in warm water (20–30 minutes) that contains approximately 0.5 to 1.0 mg psoralens per liter. UVA exposure has to be performed immediately after bathing. Alternatively, psoralen cream, usually a water-in-oil ointment containing 0.0006% 8-methoxypsoralen, is applied to a defined area of lesional skin one hour before irradiation, which has been shown to be very effective in chronic hand and foot dermatitis [66]. Therefore, cream PUVA is a good option for atopic patients with dyshidrotic eczema, in 70% complete remission has been reported. Cream PUVA is superior to bath PUVA in clearing the lesions; the moisturizing of the skin may contribute to the beneficial effect [55]. Furthermore, cream PUVA is easier in handling. The mechanisms of PUVA are less well understood compared to UVA or UVB treatment. PUVA is most effective in psoriasis, as UVA-induced DNA-psoralen photoadducts are presumed to inhibit cell proliferation [67]. This was observed at psoralen concentrations and UVA doses, which do not affect cell viability [68]. Higher doses resulted in both apoptosis and necrosis [68, 69]. The cell death of lymphocytes may be responsible for anti-inflammatory effects and for therapeutic effects on lymphoproliferative diseases, like cutaneous T-cell lymphoma. Therefore, PUVA aims to induce remissions of skin diseases by repeated, controlled phototoxic reactions. A recent publication states that PUVA reduces epidermal hyperinnervation observed in atopic dermatitis, and therefore may target the development of pruritus [70]. PUVA will be only recommended for severe atopic patients, in most cases preference will be given to other phototherapeutic regimens.

## 4. Extracorporeal Photopheresis (ECP)

Peripheral blood mononuclear cells are acquired from patients, exposed to UVA and psoralens (8-methoxypsoralen) in an extracorporeal irradiation device and are reinfused [71]. Alternatively, 8-methoxypsoralen may be taken orally before treatment. Extracorporeal photopheresis was initially developed for the treatment of cutaneous T-cell lymphoma, in particular Sézary syndrome. However, photophoresis proved to be also effective in (auto)immune-mediated diseases [72–75]. Due to its immunosuppressive/modulatory capacity, photophoresis was applied experimentally in selected patients with atopic dermatitis and in small studies and turned out to be effective in this indication when performed in four-week intervals. However, ECP had to be combined with topical corticosteroids to control disease activity [76]. Two-week intervals led to a significant improvement of the lesions, a decrease in the overall skin score as well as serum ECP concentrations could be noted [77]. High levels of total IgE turned out to be a predictor of negative outcome in ECP [78]. Beyond, a significant therapeutic effect on the quality-of-life improvement in patients who are refractory to conventional forms of therapy [79]. Recently, long-term photopheresis (>1 year) has been demonstrated to be effective in six patients with severe recalcitrant atopic dermatitis. All patients had a partial or complete remission [80]. In summary, these findings suggest that extracorporeal phototherapy is effective in the treatment of atopic dermatitis in selected patients with severe disease, which are refractory to conventional therapies. Nevertheless, ECP is expensive and time-consuming and controlled randomized studies with sufficient numbers of patients are still not available, so that this therapy is only an option for selected patients.

## 5. Carcinogenic Risk

The induction of skin tumors after long-term PUVA (>200 treatments) is undoubted, whereas the role of UVB phototherapy in human skin carcinogenesis is less clear. In a murine study, the tumor outgrowth was enhanced by broadband UVB but not by narrowband UVB or UVA-1 [81]. In mutant mice, UVB, but not UVA, was observed to induce malignant melanoma [82]. However, UVA may also play a role in the development of malignant melanoma [83]. The long term skin cancer risk of narrowband UVB is thought to be less than that of PUVA [84]. In spite of great clinical importance, no data are available concerning the long-term risk of developing skin cancer in children undergoing narrowband UVB. Furthermore, the underlying mechanisms of UVB-induced biological actions are still not entirely clear. There is recent evidence that UVB can also reach the upper parts of the dermis, however, a for long time it was supposed that UVB can only act in the epidermis due to its lower penetration compared with UVA. UVB induces apoptosis via nuclear DNA damage and direct activation of death receptors [85] and in psoriatic lesions it was shown that the therapeutic effect of narrowband UVB is associated with the induction of apoptosis of T lymphocytes [86]. The same effect may be relevant in atopic dermatitis. UVB has an immunosuppressing effect by inhibiting antigen presentation and inducing the release of immunosuppressive cytokines. It preferentially inhibits Th1 immune responses and is even able to skew immune reactions towards a Th2 type. On first sight, this would not support a beneficial effect of UVB in atopic dermatitis, since atopy in general is regarded as a Th2 driven disease. In chronic atopic dermatitis there is, however, a shift towards a Th1 reaction which also explains why it resembles much more a delayed-type hypersensitivity response. At this stage, the immunosuppression caused by UVB should be of benefit. This may explain why chronic atopic lesions have a better response to UVB than acute lesions. Atopic skin has an altered bacterial skin flora and expresses different levels of antimicrobial peptides compared with healthy or psoriatic skin. Narrowband UVB has a significant influence on the expression of antimicrobial peptides, bacterial colonization of the skin surface, and bacterial superantigens. This pathological antimicrobial response could explain the disposition for bacterial infections in atopic skin. Narrowband UVB therapy is able to reduce skin surface bacteria, superantigen production and alter mRNA levels of antimicrobial peptides, which have been shown to trigger atopic dermatitis disease activity [87, 88].

In conclusion, in view of its efficacy, benefit-risk profile, and costs, narrowband UVB should be considered as the first-line phototherapeutic option for moderately severe atopic dermatitis [89]. However, more clinical trials are needed to investigate central issues such as carcinogenicity and effectiveness in skin diseases other than psoriasis. Taken together, narrowband UVB is a very useful additive therapy for atopic patients, which significantly reduces disease activity, extent of disease, and pruritus.

## 6. Conclusions and Perspectives

Phototherapy is still one of the major therapeutic options in atopic dermatitis and this will certainly also apply in the future. New developments, like UV-free phototherapy or excimer laser treatment might broaden the indication for phototherapy [90–92]. Nevertheless, even for conventional regimes, much more clinical research is needed to confirm potential indications and determining when and how it should be used. While the development of phototherapeutic regimens was mostly empirical in the past, recent developments are based on the increase of our knowledge on the pathogenesis of atopic dermatitis as well as in the molecular mechanisms mediating the biological effects of UV radiation [48, 93, 94]. Despite that, compared for example to psoriasis, atopic dermatitis does less reliably respond to phototherapy in general. There are still a reasonable number of patients who do not respond or even get worse under phototherapy. This may be due to the fact that atopic dermatitis is a highly complex disease in which numerous factors determine the clinical outcome and the therapeutic response. It is important, to identify criteria and parameters, which will determine whether the patients will respond to phototherapy and in particular to which type of treatment. This will permit an individually customized phototherapeutic regimen for each patient.
